# Improving Oral Health Care for Frail, Community-Dwelling Older People: Exploring Barriers and Facilitators for Interprofessional Collaboration

**DOI:** 10.1016/j.identj.2025.109346

**Published:** 2026-01-02

**Authors:** Mees H.S. de Jong, Katarina Jerković-Ćosić, Monique H. van der Veen, Vanessa R.Y. Hollaar, Linet F. Weening-Verbree, Lea Kragt, Seline (W.E.) Kok, Fred R. Rozema, Claar D. van der Maarel-Wierink

**Affiliations:** a Department of Oral Medicine, Academic Centre for Dentistry Amsterdam (ACTA), Amsterdam, The Netherlands; b Department of Public Health and Oral Health Care, Academic Centre for Dentistry Amsterdam (ACTA), Amsterdam, The Netherlands; c Department of Oral and Maxillofacial Surgery, Amsterdam UMC, University of Amsterdam, Amsterdam, The Netherlands; d Research Group Innovations in Preventive Care, University of Applied Sciences Utrecht, Utrecht, The Netherlands; e Department of Preventive Dentistry, Academic Centre for Dentistry Amsterdam (ACTA), Amsterdam, The Netherlands; f Research Group Healthy Ageing, Allied Health Care and Nursing, Hanze University of Applied Sciences, Groningen, The Netherlands; g Center for Dentistry and Oral Hygiene, University Medical Center Groningen, Groningen, The Netherlands; h Department of Paediatric Dentistry, Academic Centre for Dentistry Amsterdam (ACTA), Amsterdam, The Netherlands; i Research Group Collaborating on Oral Health, Inholland University of Applied Sciences, Amsterdam, The Netherlands; j Department of Dentistry, Radboud University Medical Center, Nijmegen, The Netherlands

**Keywords:** Aged, Dental care, Oral health, Interprofessional relations, Frailty

## Abstract

**Introduction:**

Oral health is an important aspect of overall well-being, especially for frail older people, who are increasingly ageing in place. As this population with associated complex health care needs grows, integrated and collaborative oral health care becomes more urgent. Despite its importance, oral health is often overlooked in both practice and policy. This study aims to identify barriers and facilitators at the micro, meso, and macro levels to improve interprofessional collaboration for oral health care of frail, community-dwelling older people.

**Methods:**

This qualitative study was conducted in the Netherlands and comprised two data collection methods: six semistructured interviews and five discipline-specific focus group discussions, involving a total of 38 health care professionals. Each focus group consisted of professionals from a single discipline, including oral health care professionals, general practitioners, home care professionals, pharmacists, and public health professionals. Participants represented health care professionals both within nationally recognized integrated geriatric care networks and those not yet participating in such networks. Data were analysed inductively using thematic analysis in Atlas.ti.

**Results:**

At the micro level, barriers included limited awareness, knowledge, and prioritization of oral health, unclear responsibilities, time constraints, and inconsistent referral pathways. At the meso level, participants reported insufficient standardized protocols, lack of interprofessional education, and poor digital integration of medical record systems. At the macro level, key issues were inadequate funding and reimbursement, fragmented policies, and limited support for home-based oral care. Facilitators at the micro and meso level included raising awareness and interprofessional education, practical tools and clear referral pathways, improved ICT systems, and appointing regional coordinators to strengthen multidisciplinary collaboration. At the macro level, national guidance and structural financial incentives were viewed as crucial for sustainable integration.

**Conclusion:**

In conclusion, this study, identified critical barriers and facilitators for effective interprofessional collaboration and better oral health care for community-dwelling frail older people at the micro, meso, and macro levels. Improvement requires increasing awareness and education among professionals, better integration of oral health care in multidisciplinary care networks, shared electronic medical record systems, and adequate reimbursement. These findings should inform guidelines and be validated through implementation studies.

## Introduction

Oral health remains an important aspect of overall well-being, significantly impacting the quality of life and directly and indirectly impacting other health conditions. This is particularly relevant for the older population, which is steadily growing.^1^ As life expectancy continues to rise, there is a significant shift towards an ageing population. By 2022, 20% of the Dutch population was 65 years or older, a substantial increase compared to 1990, when only 12.8% of the population was in this age group. This trend is even more pronounced among those aged 80 and older; in 1990, just 2.9% of the population was 80 years or older, while by 2022, this percentage had risen to 4.9%.^2^^,^^3^ The population of community-dwelling older people continues to grow, and approximately 25% of them are considered frail, making them particularly susceptible to health problems, including oral health issues.^4^ It is increasingly recognized as important that older people remain in their homes and communities for as long as possible, even if they require assistance. While this trend supports the autonomy and well-being of older people, it also increases the risk of oral health needs going unnoticed.^5^^,^^6^ In addition, the proportion of dentate older people is rising. In the Netherlands, more than 60% of those aged 75 and older and over 80% of those aged 65 and older still have at least part of their natural dentition.^7^

As people age, there is a noticeable shift: while visits to general health care professionals tend to increase, dental visits tend to decline.^8^ Many community-dwelling frail older people no longer visit the dentist regularly, leading to dental practices often losing track of these individuals. Oral health problems can go unnoticed and untreated without regular dental check-ups, resulting in more severe health issues.^9^^,^^10^ However, often these older people are still in regular contact with other health care professionals, such as general practitioners, home care professionals, pharmacists, and medical specialists.

This raises the question of whether nonoral health professionals should play a greater role in promoting oral health care and ensuring timely referral to an oral health care professional.^11^ This increased collaboration in primary care has been advocated for several decades. However, there remains limited implementation of collaboration between oral health care professionals and other health care professionals.^12^^,^^13^ Coordinating the activities of professionals from various backgrounds and disciplines can be challenging, yet it is essential for providing comprehensive care to frail older people. Understanding the roles and responsibilities of each professional involved in the health care and oral health care of frail older people, and establishing effective communication and collaboration strategies, are critical in overcoming these challenges.

Therefore, insight into the factors that influence it at the micro, meso, and macro levels is essential. While the role of the individual health care professional is important, organizational support is equally critical for enabling interprofessional collaboration, ensuring adequate training, and integrating oral health into daily routine care practices.^14^ Without clear policies and institutional backing, individual efforts by health care professionals are unlikely to lead to sustained improvements.^15^^,^^16^

Therefore, a practical guideline and consensus are essential in encouraging interprofessional collaboration. Due to the absence of standardized guidelines and protocols, there seems to be no consensus among Dutch health care professionals about the optimal approach to oral health care for community-dwelling older people and how this should be organized collaboratively.^10^^,^^17^ This study aims to gain insight through qualitative research into the barriers and facilitators experienced in interprofessional collaboration at the micro, meso, and macro levels. These findings will contribute to the development of a practical guideline to enhance collaboration among health care professionals and within geriatric care networks, ultimately improving the oral health and overall well-being of frail, community-dwelling older people.

## Methods

### Study design and participants

This study was conducted in the Netherlands and used a qualitative research design to explore the barriers and facilitators of interprofessional collaboration on the micro, meso, and macro level for improving oral health care for community-dwelling frail older people. The approach consisted of two data collection methods: semistructured interviews and discipline-specific focus group discussions. This research is part of ORANGE-FORCE; an initiative that focuses on improving oral health care provision and interprofessional collaboration for community-dwelling frail older people.

Six semistructured interviews, each lasting 30 to 45 minutes, were conducted with eight health care professionals who were part of integrated geriatric care networks across the Netherlands. These networks are well-established primary care collaboration initiatives for integrated geriatric care, where oral health care is generally not included, with one network being a notable exception. In addition, five discipline-specific focus group discussions were held with 30 health care professionals who were not yet part of an integrated care network. These professionals represented five distinct disciplines: oral health care professionals, general practitioners, home care professionals, pharmacists, and public health service (GGD) professionals. Each focus group consisted of six to eight participants.

Individual interviews were chosen for participants within integrated care networks to allow for in-depth exploration of complex organizational dynamics and personal experiences within existing collaborative structures. Focus group discussions were used for professionals outside these networks to stimulate interaction, exchange of perspectives, and to identify shared barriers and facilitators.

All participating health care professionals had extensive work experience, were willing to share their insights, and represented different regions within the Netherlands, ensuring a diverse range of perspectives.

### Data collection

The semistructured interviews and focus group discussions were organized digitally between August and September 2022 via Zoom or Microsoft Teams and were all audio- and video-recorded and transcribed afterwards. The semistructured interviews were done by a single interviewer, and the focus group discussions by an experienced interviewer (moderator), who was also a health care professional, and one or two assistant moderators. The assistants ensured active participation, took notes, complemented as needed, and acted as time keepers.

In the focus group discussions, the following oral health care-related topics were discussed: general information (experiences, collaboration), knowledge, roles and responsibilities, referral processes, available facilities, barriers, and facilitators. Moreover, the participants could suggest new topics. The aim was to discuss the perspectives, opinions, and experiences of health care professionals and identify the barriers and facilitators of interprofessional collaboration in oral care for frail older people. The semistructured interviews focused on specific topics regarding gaining insight in the integrated geriatric care network, oral health care as part of the network, and barriers and facilitators for the implementation of oral health care. The participants were encouraged to propose new topics or raise any additional issues of concern. An interview guide (Appendix 1) was developed before the interviews and focus group discussions. These interview guides were reviewed thoroughly by the research team.

### Data analysis

The audio recordings of the interviews and focus group discussions were transcribed verbatim, ensuring anonymity through numbering the transcripts. Transcriptions were written and analysed in the Dutch language to warrant the context and interpretations. Every word and sound was transcribed, including hesitations ‘uhm’, laugher, long pauses ‘—’, and a sigh ‘…’. Moreover, the interviewer’s words as ‘aha or mmh’ were included. If a word is not audible, it was indicated by: ‘???’. The transcripts were analysed by the author according to thematic analysis (inductive coding),^18^ using the software program Atlas.ti version 22 (Atlas.ti Scientific Software Development GmbH).^19^ First, each transcript was read and reread over again. To be immersed in the data, the author listened again to the audio and watched the video recordings. Second, sentences or paragraphs of the transcripts were labelled with codes when important to the subject of the study. Text fragments were summarized by a word or short phrase (open coding). Third, after the transcript was coded, it was explored if there were codes which had a similar meaning. These codes were merged, so the number of codes was reduced (axial coding). Fourth, themes (categories) were made according to the topics of the interview guide to put the codes in categories. If needed, a theme was added to the pre-existing topics. A theme clustered the codes that seem to share a characteristic. A theme also can have subthemes. Fifth, researchers of ORANGE-FORCE were involved and examined and refined the codes and themes to ensure validity. The process of coding consisted of adjusting of the codes throughout the entire procedure. The coding was considered finished when the researchers obtained consensus. Afterwards, codes, themes, and quotes were compared to find similarities and contrasts. The links, relationships, and patterns between themes were analysed (selective coding). From this, conclusions were drawn, and these were presented in text with anonymized quotations of the participants. Summaries were created for each focus group interview and were sent to the participants, asking whether the content accurately reflected what was discussed. Additionally, they were asked if they felt free to express their opinions during the discussion.

Themes were subsequently categorized according to whether they reflected micro-, meso-, or macro-level influences on interprofessional collaboration. This structured approach facilitated the integration of findings across all levels of analysis.

## Results

### Study design and participants

Six interviews and five focus group discussions were conducted, involving a total of 38 health care professionals from various disciplines, including 11 oral health care professionals, eight general practitioners, six home care professionals, five pharmacists, and eight public health service (GGD) professionals. Of these, eight participants were already part of an integrated care network, while 30 were not. Demographic information is summarized in Table 1.

**Table 1:** Characteristics of health care professionals participating in the semistructured interviews and focus group discussions.

Discussion/interview	Participant	Gender (female/male)	Age	Years of work experience	Profession
**Focus group discussion 1:**	1	F	43	17	Pharmaceutical scientist
**Pharmacists**	2	F	Unknown	28	Pharmacist
	3	M	49	24	Pharmacist
	4	F	40	14	Pharmacist
	5	M	45	20	Pharmacist
**Focus group discussion 2:**	1	F	63	10	Dental hygienist
**Oral health care professionals**	2	F	40	Unknown	Dental hygienist
	3	M	59	31	Dentist
	4	M	34	6	Dentist
	5	F	41	17	Dental hygienist
	6	M	60	35	Dental technician
	7	F	42	18	Dentist
	8	F	42	14	Dentist
**Focus group discussion 3:**	1	F	30	5,5	Health promoter
**GGD professionals**	2	F	65	45	health promoter
	3	F	51	1	Student ‘healthy ageing’
	4	F	61	33	Health promotion officer
	5	F	44	3	Health promoter
**Focus group discussion 4:**	1	F	62	Unknown	District nurse
**Home care professionals**	2	M	53	Unknown	Process manager and district nurse
	3	F	64	25	Nursing assistant
	4	F	60	9,5	Nursing assistant
	5	F	48	Unknown	Nursing assistant
	6	F	23	Unknown	District nurse support
**Focus group discussion 5:**	1	F	62	22,5	General practitioner
**General practitioners**	2	M	36	3	General practitioner
	3	M	63	35	General practitioner
	4	F	62	20	General practice nurse
	5	F	46	15	General practitioner
	6	F	40	Unknown	Dentist
**Semistructured interviews with professionals from integrated networks**	1	F	Unknown	Unknown	Coordinator elderly care
	2	F	Unknown	Unknown	General practitioner specialised in geriatric care
	3	F	Unknown	Unknown	General practitioner specialised in geriatric care
	4	F	Unknown	Unknown	Coordinator elderly care
	5	F	Unknown	Unknown	Chain director dementia care
	6	F	Unknown	Unknown	Nurse practitioner
	7	M	Unknown	Unknown	Geriatric dentist
	8	F	Unknown	Unknown	Geriatric dentist

### Analysis and findings

Thematic analysis of the data identified five themes. These themes were further divided into a total of 36 subthemes from the interviews and 34 subthemes from the focus group discussions. Identified themes include ‘Importance and Awareness of Oral Health’, ‘Interprofessional Collaboration and Integration of Oral Health Care’, ‘Knowledge, Skills, and Experiences’, ‘Organization, Implementation, and Facilities’, and ‘Signalling Screening and Prevention’. Each theme and its corresponding subthemes are comprehensively detailed in Tables 2 and 3. Each theme is explained individually and illustrated using quotes from the data. Barriers and facilitators across are presented in Table 4, Table 5, Table 6.

**Table 2:** Framework of the focus group discussions.

Theme	Subtheme	No. quotes	Total no. quotes
Importance and awareness of oral health	References to the importance of attention to oral health	30	
	References to the importance of awareness or attitudes among professionals/patients	22	
	Tools, campaigns, and actions to increase knowledge or awareness	26	
	References to setting priorities, sense of urgency, and policy	12	
	Practical or structural barriers to improving oral health care	20	
	Embedding in care plans and collaboration among professionals	10	
			120
Interprofessional collaboration and integration of oral health care	References to the integration of oral health into general care, public health policy, or multidisciplinary approaches	27	
	Statements about the distribution of responsibilities in collaboration, including roles of health care professionals	27	
	References to consultations, communication, or referrals between health care professionals	34	
	Education, information-sharing, and collective awareness within care teams	11	
	Barriers to interprofessional collaboration or integration	75	
	Facilitating factors, successes, or opportunities in collaboration	25	
	Digital or practical tools for collaboration or communication Specific referrals or uncertainties about who should refer and why	9 11	
			219
Knowledge, skills, and experiences	General knowledge of oral health and oral health care in older people, including recognition of side effects and risks	17	
	Understanding and use of guidelines related to oral health care in older people	9	
	Practical experience or skills, especially with frail older people	7	
	Training, continuing education, and initiatives to strengthen knowledge or skills.	16	
	Lack of knowledge, failure to apply existing knowledge, or barriers to sharing knowledge	31	
			90
Organization, implementation, and facilities	References to government policies, public health approaches, local initiatives, and strategies related to oral health.	16	
	Barriers in implementation or variation between public health services, colleagues, or institutions	60	
	Aspects related to reimbursement, subsidies, budgets, and funding as a condition or barrier	13	
	Practical aspects of delivering oral health care in daily practice	24	
	Integration of oral health care into existing care structures such as ADLs, intake procedures, community nursing, or institutional care	16	
	Laws, regulations, privacy legislation, and ethical dilemmas related to care delivery	9	
	Positive elements that support the organization or implementation of oral health care	12	
			150
Signalling, screening, and prevention	References to actively monitoring patients, identifying those who have dropped out of care, and using protocols or systems	25	
	References to signs of problems, observations by caregivers, or tools to recognize issues at an early stage	16	
	Informal caregivers and family members as key players in the detection and communication of oral health concerns	9	
	Responsibility for follow-up, scheduling appointments, and ensuring continuity of care	9	
	Prevention through structured oral care routines and attention to at-risk groups	14	
	The role of pharmacists in identifying issues, reviewing medications, and making referrals	13	
	Barriers such as lack of time, frailty of older people, or absence of tools and protocols	28	
	Raising awareness of at-risk groups, providing targeted education, and identifying risk factors	10	
			124

**Table 3:** Framework of the semistructured interviews.

Theme	Subtheme	No. quotes	Total no. quotes
Importance and awareness of oral health	Awareness of the importance of oral health among patients, family members, and informal caregivers	27	
	Oral health is not a priority and an overlooked subject	7	
	Creating awareness that the mouth is an integral part of overall health	9	
	Importance of daily oral hygiene care	3	
	Home visits by a dentist may lower the barrier for older people to seek care	2	
	Education and repetition about oral health increases awareness	9	
	Raise awareness from the moment the relationship between care provider and patient begins	4	
	Information sources to increase awareness	10	
	Fostering enthusiasm rather than imposing mandates	7	
	Improving self-reliance of older people	3	
			81
Interprofessional collaboration and integration of oral health care	Strengthening collaboration and establishing clear lines of communication	35	
	Establishing clear rules and agreements facilitates easy collaboration	9	
	Interprofessional education by means of workshops/training/videoclips	18	
	General references to interprofessional collaboration	7	
	Time and workload are a barrier for making contacts	12	
	Integrating oral health care as a component of general health care	42	
	Practice nurses and general practitioners addressing oral health concerns	4	
	The need for a geriatric dentist or an ambulant dentist in a geriatric care network	10	
	National or regional consultation point for oral health care	7	
	Need for a protocol/guideline/tool on oral health care for frail older people	15	
			159
Knowledge, skills, and experiences	Knowledge about what to do when there are complications	9	
	Significant experience and skill needed to conduct an oral examination	9	
	Performing a check-up can be challenging	2	
	Anticipating potential problematic situations	3	
	Barriers, including boundaries, obtaining consent, and older people with dementia	10	
			33
Organization, implementation, and facilities	Suggestions for ICT applications	13	
	Including oral care as an item on the problem list or checklist	12	
	Suggestions for tools, identification of needs, or recognition of missing resources	31	
	Oral health professionals are affiliated with small organizations, challenging collaboration	5	
	Frequent turnover and competition between health care professionals	1	
	Dental care costs of older people	5	
			67
Signalling, screening, and prevention	Identifying and referring frail older people to the geriatric care network	40	
	Advance care planning meetings are important to establish the care plan	13	
	Clarifying the role of the dentist	8	
	Clarifying the role of the case manager	9	
	First signals, problems, and making a referral to a specialist	12	
			82

**Table 4:** Barriers and facilitators on the microlevel.

Barriers	
Time constraints in general practice	General practitioners often cite the lack of time as a significant barrier to conducting oral examinations.
Professional embarrassment	There’s an underlying issue of professional embarrassment among dental care professionals when it comes to referring patients with complex conditions such as dementia or Parkinson’s disease.
Lack of referral pathway knowledge	A crucial barrier is the widespread uncertainty among health care professionals about the appropriate pathways for referring patients with oral health problems.
More inclined to visit general practitioners	Older people are more inclined to visit the general practitioner instead of oral health care professionals, even if there are oral problems.
Resistance to referral	There’s a notable resistance among older people to being referred to oral health care professionals.
Lack of motivation	The prioritization of oral health care for frail older people is often lacking among oral health care professionals.
Lack of awareness	Both patients and nonoral health care professionals frequently exhibit a lack of awareness about the critical importance of oral health.


Facilitators	
Regular education and training	Ongoing educational programs for both oral health care professionals and general health care providers.
Role clarity for dentists	Clearly defining the responsibilities and roles of dentists in the care of frail older people.
Involvement of general health care professionals	Encouraging nondental health care providers to play a role in recognizing and addressing oral health issues.
Proactive problem identification	Implementing strategies for early detection of oral health problems among older people.
Respect for boundaries	Maintaining a sensitive approach to oral health care, especially for patients with cognitive impairments, to ensure their comfort and dignity.
Train-the-trainer programs	Developing programs where trained individuals can further educate others within the health care system about oral health care.
Raising awareness	Utilizing brochures, websites, warning labels on medications, and information screens in waiting rooms to educate patients about the importance of oral health.

**Table 5:** Barriers and facilitators on the meso-level.

Barriers	
Lack of communication	Lack of streamlined communication channels between oral health professionals and other health care providers hinders effective care coordination.
Resource limitations	Health care facilities often face resource constraints, including inadequate staffing and insufficient equipment for comprehensive oral health care.
Inadequate training	There’s a gap in specialized training for health care providers on the importance of oral health, leading to underprioritization in patient care plans.
Challenges for home-based oral care	Home-based oral care is demanding: it involves physical strain, time constraints, and insufficient equipment; often extends beyond regular hours; and faces poor reimbursement and challenges in securing insurer approvals
Accessibility of professionals	Contacting oral health care professionals poses difficulties, impacting the efficiency of referral and consultation processes.
Knowledge gaps	There is a noticeable deficit in oral health knowledge among nonoral health professionals, contributing to underestimation of oral health’s importance in overall care.
Workload and reimbursement issues	The high workload experienced by general practitioners and pharmacists, coupled with insufficient reimbursement for integrating oral health into practice, reflects systemic undervaluation of oral health care.
Funding challenges	Securing funding for oral health initiatives, particularly within GGD organizations, remains a significant obstacle.


Facilitators	
Integrated care programs	Participation of oral health care professionals in integrated care programs encourages a holistic approach to patient health, emphasizing the importance of oral care.
Pharmacist initiative	Pharmacists taking proactive steps, such as warning labels on medicine, can highlight the impact of medications on oral health.
Oral health in care plans	Ensuring oral health is a standard component of general care plans emphasizes its importance in comprehensive health care.
Examination equipment and visual information	The availability of proper tools and informative materials in care settings aids in early detection and patient education.
ICT programs for data sharing	Implementing ICT solutions facilitates better coordination and communication among health care providers.
Multidisciplinary team meetings	Utilizing modules with cases in team meetings encourages collaborative problem-solving to complex cases and can also be organized regionally to enhance interprofessional collaboration.
Leadership in coordination	A designated regional coordinator can spearhead multidisciplinary efforts, ensuring consistent focus and action on oral health issues.
Communication platforms	Tools like chat function groups facilitate swift, direct communication among care providers, enhancing coordination.
Tele-dentistry	This technology offers a solution for remote consultation, aiding professionals in situations of uncertainty or when seeking specialized advice.
Continued education	Webinars and educational programs promote interprofessional collaboration and raise awareness about the significance of oral health among all care providers.
Screening questionnaires	Implementing simple screening tools in pharmacies and general practices helps identify potential oral health issues early on.

**Table 6:** Barriers and facilitators on the macro-level.

Barriers	
Legislative focus	Current laws primarily concentrate on the youth, leaving older populations with inadequate oral health care coverage.
Budget constraints in GGD organizations	Financial limitations within GGD organizations lead to a diminished emphasis on oral health.
Insurance coverage and financial considerations	The structure of insurance policies often excludes comprehensive dental care for people, making it less accessible and affordable.
Administrative burden for oral health care professionals	Burdensome processes to obtain authorization from insurers for providing home-based care reflect inefficiencies in the system.
Lack of oral care in home care plans	The systematic omission of oral health from home care plans indicates a broader oversight in care coordination, pointing towards the necessity for integrated care approaches.
Lack of ICT integration for oral health professionals	The exclusion of oral health professionals from ICT facilities used in general health care contributes to fragmented care delivery.
Limited awareness and recognition of guidelines	The KIMO and KNMT practical guidelines are not widely known among health care professionals, and their added value is often not recognized, indicating a gap in professional education and guideline dissemination.
Lack of dedicated oral health policy	Variability among GGD organizations in dedicating policies to oral health reflects a broader inconsistency in prioritizing oral health care, leading to disparities in service delivery.
Oral health not a central priority	The absence of central support for oral health at GGD-GHOR highlights a systemic undervaluation of oral health care within public health agendas.
Insufficient reimbursement for home consultations	The lack of adequate reimbursement models for oral health care consultations at home presents financial barriers to accessing necessary care for older people.


Facilitators	
ICT program implementation	Facilitating data sharing among health care professionals to streamline patient care and improve outcomes.
Access to national records	Ensuring health care providers can access national electronic patient records and the National Exchange Point (Landelijk Schakelpunt) for comprehensive patient history.
Integration into chronic care programs	Embedding oral health into existing coordinated care programs to improve access, financing, and interprofessional collaboration.

These five themes relate to different levels of the health care system: Importance and Awareness of Oral Health and Knowledge, Skills, and Experiences primarily reflect microlevel factors; Interprofessional Collaboration and Integration of Oral Health Care spans both the micro and meso levels; Organization, Implementation, and Facilities mainly reflects meso- and macro-level factors; and Signalling, Screening, and Prevention encompasses microlevel processes with meso-level organizational aspects.

### Importance and awareness of oral health

The findings show a significant difference in the prioritization of oral health care among general health care professionals. Both time constraints and a lack of affinity for providing care to frail older people contribute to this. One dentist said, ‘Many people have no affinity with providing care to frail older people. This might also apply to me’ (1.216). This was similarly observed among other health care professionals. For instance, a general practitioner acknowledged, ‘We do not structurally think about or work on oral health enough’ (2.109), while a pharmacist admitted, ‘We pay little attention to oral health, perhaps because we know too little about it’ (4.89). Similarly, a district nurse explained, ‘Oral health care is the last thing you think of at first’ (5.164), and another health care professional added, ‘It’s often the last thing on the agenda; we focus more on wounds or other immediate concerns, and oral health tends to get forgotten unless it’s brought up specifically’ (5.54).

This lack of prioritization is further complicated by challenges in involving clients in oral health care, as one professional noted, ‘Clients are more reluctant to allow us to contact a dentist compared to a general practitioner. It’s harder to act on oral health concerns because clients often do not see it as important’ (5.600). Additionally, a district nurse pointed out, ‘Daily health care often focuses on washing, even daily washing, but daily oral care is actually much more important’ (5.186).

A recurring underlying cause mentioned by participants is the high workload in health care, which limits the time available for preventive or nonurgent care. This workload is not merely an individual challenge but a systemic issue shaped by health care policies and organizational priorities. One dentist stated, ‘We are extremely busy, and things are sometimes forgotten, especially when no annual policy or protocol is in place’ (1.110). Another professional emphasized that limited reimbursement structures and the lack of national prioritization further diminish attention for oral health: ‘Oral health is not a priority within the national health agreement, so we don’t get central support for it’ (3.96).

Financial constraints also play a role, especially for older people themselves. A general practitioner explained, ‘Older people experiencing severe pain may refuse to seek treatment due to concerns about the associated financial costs’ (7.98). These barriers at the policy and organizational levels contribute to the neglect of older patients’ oral health needs.

However, there are initiatives that have been successful in raising awareness regarding the importance of oral health. Efforts by GGD professionals, such as disseminating information through newsletters and educational sessions, to increase awareness among both general health care professionals and older people. As explained by a GGD professional, ‘We try to involve and stay connected with dentists by creating a newsletter and setting up a project group’ (3.140). Similarly, awareness programs such as e-learning modules and short educational courses were mentioned as effective tools to remind health care professionals of the importance of oral health and increase their confidence in addressing it. As one district nurse described, ‘The e-learnings remind us of the importance of oral care and help us stay aware’ (5.384).

Empowering older people to take charge of their own oral health and motivating health care professionals through the sharing of success stories were identified as effective strategies. As one professional stated, ‘Sharing success stories can inspire and increase enthusiasm, rather than viewing oral health care as an obligation’ (10.47).

Finally, some participants described early examples of integrating oral health into broader chronic care programs. One professional explained, ‘We initially started by identifying frail older people through case-finding. Over time, this process has become more organic, with oral health being considered within existing programs, such as diabetes care. By incorporating it into broader chronic care programs, we can address more than just specific conditions’ (8.22).

### Interprofessional collaboration and integration of oral health care

There are several challenges with the integration of oral health care into primary care. These include the previously mentioned high workloads and the absence of routine oral health care practices within general practice, home care, and pharmacy. Additionally, individual health care professionals face difficulties in coordinating and aligning their approaches. An elderly care coordinator noted, ‘Establishing uniform agreements is a challenging task. Consistency in these agreements would greatly ease implementation’ (7.43).

A recurring barrier is time constraints, already mentioned as a key factor influencing the prioritization of oral health, also hinder the integration of oral health care into daily routines and collaboration between professionals. A general practitioner stated, ‘We are simply overloaded with tasks. Integrating something new, like oral health care, means more investment in time and effort, which is already scarce’ (2.377). This opinion was shared during a focus group with GGD professionals: ‘You give your presentation, everyone is enthusiastic, and then you close the day with: “Yes, but we just don’t have time for it”’ (3.47).

In addition to time constraints, a lack of organizational structures and communication channels hampers integration. One dentist stated, ‘We have contact from time to time, but not about older people or anything like that’ (1.144). Another general practitioner added, ‘We say very easily, “Just go to your dentist,” or the dentist says, “See your GP,” without really knowing who we are referring to or collaborating with’ (9.38). This absence of structured communication and referral pathways reinforces fragmentation between disciplines.

Resistance to accepting standardized protocols is another barrier. One general practitioner noted, ‘If we introduce fixed items for oral health in multidisciplinary meetings, it might feel like a checklist, which many resist’ (8.76). At the same time, the absence of such protocols leads to inconsistency and reliance on individual initiative. A professional emphasized, ‘Establishing a framework for physical examination that includes oral health has proven to be effective’ (8.50). Integrating oral health into existing assessments helps normalize it within routine care.

Another barrier experienced within home care was addressing oral health with clients, ‘It feels awkward to ask a client if you can watch them brush their teeth or look in their mouth. It’s different from asking about washing or dressing’ (5.231). Similarly, a GGD professional stated, ‘I found it very difficult to get through to care workers; they didn’t really want to deal with oral care and found it unpleasant’ (3.58).

Barriers regarding the lack of established referral pathways and routine communication between health care professionals and dentists are particularly evident. A pharmacist stated ‘We are not used to having contact with oral health care professionals. The general practitioner is under that button, but the dentist is not’ (4.164), while a general practitioner added, ‘What I really miss is a link with a dentist in our setup, and I have been missing that for a long time’ (11.83).

System-level challenges, such as the absence of integrated ICT systems, also hinder collaboration. Several participants emphasized the potential of shared electronic records and digital communication tools. One professional explained, ‘If our ICT systems integrated dental visits and medications, it would streamline communication and referrals’ (2.425). Another added, ‘Our digital platform allows professionals from different disciplines to exchange information, including oral health professionals’ (8.28). Broader inclusion of oral health professionals in these systems, supported by regional collaborations and shared resources, was seen as key to sustainable integration (1.596).

Despite these barriers, a lot of health care professionals showed a willingness to integrate oral health care into their daily practices, recognizing the importance of interprofessional collaboration. One health care professional emphasized, ‘Collegial contact between health care professionals is essential. That is how you establish low-threshold contact for consultation’ (6.45). Some shared that incorporating oral care as part of routine tasks aids with this integration, ‘At our team, oral health is just part of the daily ADL tasks, it’s routine for us’ (3.438). Others stated practical solutions to support this integration, ‘Simply placing oral care items like toothbrushes in sight ensures that it becomes a natural part of the daily care routine’ (5.513).

### Knowledge, skills, and experiences

The difference in educational backgrounds and organizational structures among home care professionals results in inconsistent oral health care provision. A dentist stated, ‘Home care professionals are the least predictable group in health care due to the diversity in education and organization’ (1.174). This diversity contributes to varying levels of competence and attention to oral health between teams and regions. Something similar is observed among pharmacists, particularly in how the severity of oral side effects is assessed. These side effects are often weighed against other medication-related side effects, which can influence their prioritization. As one pharmacist noted, ‘If we could properly estimate the severity of complications, we would prioritize it earlier. Maybe we underestimate the problem’ (4.193).

Lack of knowledge about oral health among nonoral health care professionals is a significant barrier. A general practitioner said, ‘At this moment, on our side there is a lack of knowledge among team members’ (9.14). Similarly, a home care professional noted the limited training they receive ‘We don’t really receive specific training about oral health. It’s sometimes mentioned briefly in other trainings, but it’s not a dedicated focus’ (5.390). Another general practitioner admitted, ‘I don’t feel confident enough to recognize oral health problems unless it’s very severe’ (2.365).

Several participants emphasized that this lack of knowledge is reinforced by limited familiarity and use of existing guidelines, such as those developed by KIMO and KNMT. A dentist acknowledged, ‘Well, for me, I do not know if the guideline has added value. There is no special information for me in it’ (1.206). This indicates that guidelines are not yet embedded in daily practice and that better dissemination and alignment with professionals’ needs are required.

The intimate nature of oral examinations presents an additional challenge, especially in respecting the autonomy of frail older people. A professional emphasized, ‘It is important to assess whether you are allowed to examine their mouth, as many may not consent to it’ (6.113). This requires additional skills and experience to handle such situations sensitively and effectively.

Education and training initiatives were identified as key facilitators for improving oral health care. An oral health care professional suggested, ‘You can teach them to recognize affected teeth and inflamed gingiva’ (6.105). A home care professional emphasized the value of education, stating, ‘Raising awareness through education is crucial. When awareness increases, you begin to consider oral health issues in relation to other health concerns’ (7.85).

Educational efforts were also discussed in a broader interprofessional context. Pharmacists, for instance, proposed including oral health information in existing consultation structures. ‘Developing standard pharmacotherapeutic consultation modules on oral health could make it easier to integrate oral health topics into regular care discussions’, one pharmacist explained (4.223). Another added, ‘Providing pharmacists with patient education materials, such as flyers or digital content, can improve patient engagement’ (3.178). Local professional meetings, such as pharmacotherapeutic consultations (FTOs), were considered valuable for exchanging knowledge and aligning approaches between disciplines (4.201).

Sharing knowledge and materials between organizations, such as GGDs, was viewed as important but not always easy. One GGD professional said, ‘All our films and materials should be available for other GGDs to use; it’s about sharing knowledge’ (3.178). Another noted, ‘I love the magazine you made. I want to copy it, but it is not that easy’ (3.84). These examples underline the need for better infrastructure to facilitate knowledge-sharing and ensure consistency across regions.

Finally, digital solutions such as tele-dentistry were mentioned as potential tools for enhancing access to expertise. A district nurse explained, ‘Using tele-dentistry or even just photos and videos could help us get advice from oral health care professionals without needing a physical visit’ (5.637). Integrating oral health into medication review protocols was also suggested as a simple way to increase attention for oral health in older people (4.67).

### Signalling and screening of oral health problems and prevention

Oral health screening is almost completely absent in the general practice, where it is particularly important given the frequent contact with frail older people. A nurse practitioner said, ‘No, I do not examine the mouth. Due to a lack of time, but that is kind of a lame argument’ (2.317). This is also evident in home care settings, where daily oral hygiene problems are often overlooked, and even when limitations are noticed, no follow-up action is taken. A district nurse noticed, ‘If you notice someone is bad with their hands, then I wonder if brushing their teeth would go well’ (5.260). Additionally, client resistance to oral care further complicates this, one nursing said, ‘Some clients have a lot of resistance to and close their mouth firmly together’ (5.663).

The unclear role of dentists in the care of frail older people results to the neglect of both the patients and the dentist. A professional remarked, ‘It’s easy to forget an older adult. The older adult forgets to visit the dentist, but the dentist also forgets his older patient’ (11.27). This lack of clear responsibility highlights the importance of involving general health care professionals in the early identification of oral health problems.

Additionally, even when there is willingness among many professionals, the absence of systematic screening tools and routines in primary and home care settings limits early detection. A district nurse noted, ‘We rarely have contact with dentists or dental hygienists. If there’s a problem, it’s often left to the family to manage’ (5.350). Participants emphasized that without clear procedures or communication pathways, early warning signs are easily missed. Organizational support and coordination with oral health care professionals are essential to ensure follow-up and prevention.

One nurse suggested, ‘During our regular visits, including questions about oral health as part of the standard assessment seems achievable’ (9.4). The potential impact of routine oral health checks was emphasized, as it was noted that ‘asking three simple questions about oral health during consultations with frail older patients could make a significant difference’. A general practitioner confirmed that this approach is ‘feasible and easy to implement’ (2.215).

Integration of oral health into existing preventive programs was frequently mentioned as an effective strategy. A public health professional shared, ‘We often connect with fall prevention programs to promote oral health during their sessions’ (3.62). Such cross-program integration normalizes oral health as part of general prevention and supports a broader health perspective.

Digital tools were also mentioned as promising innovations to enhance access and continuity of care. A general practitioner explained, ‘Tele-dentistry can reduce the need for unnecessary consultations, especially for older people who cannot visit a dentist easily’ (2.485). Photographs and digital consultations were considered useful for identifying problems early, especially in home care.

Regional collaboration further supports better screening and referral. One professional noted, ‘We started by creating local networks with home care staff, GPs, and pharmacists around older people’ (3.134). Another added, ‘We have a network group with dentists and dental hygienists, so we can easily reach out if a client has a problem. It’s a great support system’ (5.344).

### Organization, implementation, and facilities

Beyond individual and interprofessional efforts, organizational and policy frameworks play a crucial role in how oral health care is implemented and sustained in the care for home-dwelling frail older people. Changes in these frameworks have negatively impacted the time and funding available for oral health care. As explained by a public health professional, ‘Legislative changes, such as the Public Health Act, removed the requirement for GGD organizations to employ dentists, making oral health a discretionary priority rather than a mandated one’ (3.101).

There are limited options for providing oral health care at home to older people who can no longer visit a dental practice. This issue is further exacerbated by administrative burdens. A dentist noted, ‘Reimbursement for home visits is necessary, as they require more time, and, as they say, nothing comes for free!’ (8.70). Insufficient policy support further burdens professionals. The physically demanding nature, inadequate compensation, and lack of proper equipment for providing care at home make this even more challenging.

At the national level, oral health remains insufficiently prioritized in preventive care agreements, limiting central support and funding. One public health professional explained, ‘Most nurses and general practitioners do not yet include oral health in their consultations, even though it’s important for patients with diabetes or heart problems’ (3.50). A home care professional emphasized a challenge within home care, ‘There is no uniform protocol for oral health assessments during intake, which results in inconsistent attention to oral hygiene’ (5.182). Structural barriers in data sharing also limit integration. ‘The systems between dental care and other health professionals are not connected, so dentists lack access to patients’ medication history’ (4.146).

## Discussion

The findings of this study highlight several critical aspects influencing interprofessional collaboration in oral health care for frail, community-dwelling older people. At the micro level, a lack of prioritization and awareness of oral health care was noticeable among health care professionals, worsened by time constraints and limited affinity for providing care to this population among oral health care professionals. Microlevel barriers were also reflected in gaps in knowledge and skills and the absence of clear referral pathways. At the meso level, substantial barriers were identified in integrating oral health care into primary care, including the absence of standardized protocols, limited digital integration, and challenges in interprofessional communication and coordination. At the macro level, insufficient prioritization of oral health in national health care policies and funding structures leads to inadequate insurance coverage and financial constraints for both patients and professionals.

Despite these barriers, there is recognition among health care professionals of the importance of interprofessional collaboration and a willingness to improve oral health care. Several facilitators were identified. At the micro level, joint training sessions, shared educational resources, and increased awareness among health care professionals strengthen confidence, skills, and early recognition of oral health problems. At the meso level, digital solutions, such as shared electronic medical records and tele-dentistry, can improve communication, coordination, and access to expertise. Integrating oral health care into existing chronic-disease management and preventive programs offers a practical route towards sustainable implementation. For example, in the Netherlands, chronic disease management programs such as the diabetes coordinating care program bring together general practitioners, specialists, and other health care providers to offer comprehensive care.^20^ Including oral health into such programs could provide a sustainable framework for prevention and early intervention.^21^ Additionally, appointing a designated regional coordinator could help facilitate multidisciplinary efforts, ensuring a more structured and consistent focus on oral health within integrated care networks. At the macro level, establishing national guidance and long-term policy support for interprofessional collaboration, along with creating structural financial incentives, can enable more sustainable integration of oral health care into routine primary care.

In line with these findings, other studies also show this lack of prioritization, awareness, and knowledge. The Sûne Mûle project, which emphasizes the importance of preventive oral care and raising awareness among home care nurses and community-dwelling older people to maintain their oral health and overall well-being.^22^ Furthermore, Niesten et al^23^ demonstrated in a qualitative study that many frail older people do not recognize the importance of visiting a dentist. This is increased by the belief among many older people that dental visits are unnecessary if they are edentulous.^24^^,^^25^ Financial issues also play a role, making older people more likely to visit their general practitioner rather than an oral health professional.^26^

Lack of motivation and awareness are often mentioned to be barriers. Similar to this study, it was previously suggested that interprofessional education can improve collaboration among health care professionals.^27^^,^^28^ During the discussions and interviews in this research, it became clear that the knowledge among health care professionals regarding oral health care is often insufficient; this is also seen in previous research that highlighted the poor understanding of oral health among nonoral health care professionals as a barrier to effective collaboration.^29^^,^^30^ This emphasizes the need for improvement through education and training initiatives, which have been shown to contribute in better oral health care.^31^, ^32^, ^33^

A crucial issue frequently mentioned is the limited time available to health care professionals. Previous literature also identifies this as a significant barrier that needs to be addressed. Clear roles, tools to quickly differentiate between the severity of oral health issues, and strategies for early detection could help with this problem.^34^

Previous research supports the importance of system determinants, showing that weak national health policies and limited resources remain major barriers worldwide, while integration of oral health into national programs and financing primary health care that is tailored to the needs of older people, and ensuring the availability of oral health services are identified as important facilitators.^35^, ^36^, ^37^ The importance of digital integration and education is also emphasized in the literature, with electronic medical records and tele-dentistry facilitating interprofessional collaboration and continuity of care.^38^, ^39^, ^40^, ^41^

However, this study has limitations that should be acknowledged. The study is based on qualitative data, which, while providing deep insights, may not be generalizable across all settings or populations. The findings are also influenced by the specific context of the Dutch health care system, which may limit their applicability in other countries.

However, this study has limitations that should be acknowledged. As a qualitative exploratory study, the aim was not to generate generalizable findings but to provide in-depth insights into interprofessional collaboration in oral health care. The findings are therefore context-dependent and shaped by the structure of the Dutch health care system. Further research is needed to examine whether the barriers and facilitators identified in this study are experienced more broadly across other regions, settings, and health care systems.

Future research should focus on translating these insights into practical tools and guidelines that health care professionals can use to improve collaboration and integrating oral health into daily practice. The insights from this study offer a basis for informing policy development and will be discussed with key stakeholders who can influence the policy framework. Elements that could be incorporated into a guideline include clearly defined roles and responsibilities, practical tools for daily integration of oral health, referral pathways, and access to relevant knowledge resources.

In conclusion, this study identifies critical barriers and facilitators across the individual, organizational, and policy levels that influence interprofessional collaboration in oral health care for frail, community-dwelling older people. Based on this study, effective improvement of interprofessional collaboration in oral health care depends on raising professional awareness, strengthening organizational and digital infrastructure, and ensuring supportive reimbursement and policy frameworks. These findings provide a comprehensive foundation for developing a practical guideline to improve integration of oral health care into daily practice.

## Ethics statement and informed consent

Ethical approval was obtained from the Institutional Review Board (IRB) of the Academic Centre for Dentistry Amsterdam (ACTA). Registration number: 2022-31823/2022-45572. All participants provided informed consent. Data was handled confidentially and anonymously.

## Author contributions

This work was carried out in collaboration among all authors. MJ contributed to the manuscript by drafting the majority of the text and incorporating received feedback. CW and MV also contributed significantly to the writing process, provided valuable insights regarding the study’s design, and offered critical feedback. KJ, FR VH, LW, WK contributed to the writing, gave crucial feedback, and provided strategic advice on the study’s design. All authors read and approved the final manuscript.

## Funding

The collaborative project is cofunded with a PPS surcharge provided by Health-Holland, Top Sector Life Sciences & Health, to encourage public-private partnerships.

## Conflict of interest

All authors have no conflict of interest to declare.
